# Structural characterization of polysaccharides recovered from extraction residue of ginseng root saponins and its fruit nutrition preservation performance

**DOI:** 10.3389/fnut.2022.934927

**Published:** 2022-08-01

**Authors:** Jing Sun, Xinyu Zhong, Dandan Sun, Xinxin Cao, Fan Yao, Lingling Shi, Yujun Liu

**Affiliations:** ^1^National Engineering Laboratory for Tree Breeding, College of Biological Sciences and Biotechnology, Beijing Forestry University, Beijing, China; ^2^Shandong Academy of Chinese Medicine, Jinan, China

**Keywords:** ginseng residue polysaccharide (GRP), structure characterization, antioxidant activity, GRP coating, fruits preservation

## Abstract

Polysaccharides recovered from extraction residue of ginseng root saponins, i.e., ginsenosides-extracting residue polysaccharides (GRP), were separated into two fractions, GRP-1 and GRP-2. Fourier infrared and nuclear magnetic resonance spectra, as well as high-performance liquid chromatography and gel permeation chromatography measurements, showed GRP-1 was composed of mainly starch-like glucans and GRP-2, relatively a smaller portion, was a mixture of heteropolysaccharides composed of starch-like glucans, rhamnogalacturonan-I pectin, and arabinogalactans, and they had similar molecular weights. These results proved that the structure of GRP was not destroyed and GRP still maintained strong antioxidant activities. In addition, GRP coating on surfaces of fruit slowed their deterioration and maintained their nutritional effects. Correlation and PCA analyses on various quality and antioxidant parameters supported the above findings and a possible mechanism in fruit preservation was then proposed. Knowing the structural features and bioactivities of GRP gives insights into its application. Specifically, GRP served as an environmentally friendly coating that can be used to preserve the nutrients and other quality indicators of strawberries and fresh-cut apples, paving the way for future new approaches to food preservation using polysaccharides or other natural products.

## Introduction

Edible coatings mainly made of polysaccharides, proteins, resins, and lipids (1), are new environmentally friendly packaging. Recently, polysaccharides-based coatings have attracted increasing attention in that they can instead of chemical additives maintain the nutrients and other quality indicators of fruits by controlling the respiration rate, preserving rigidity, and acting as antioxidants (2). Several applications of polysaccharides-based coatings have been reported to be effective in fruit preservation. For instance, the aloe vera gel coating reduced browning and maintained the postharvest quality of the litchi fruit (3), and the chitosan-pectin-cinnamaldehyde coating extended the shelf-life of fresh-cut cantaloupe (4). The development of packaging with natural products, such as polysaccharides, can be considered an innovative strategy not only to prolong the shelf-life of food but also to attract the consumer and impact the marketing of products.

Like ginsenosides, ginseng polysaccharides, also a kind of important active component, possess various pharmacological effects including antioxidant (5), antifatigue (6), and improvement of immune function (7). Compared with polysaccharides of other sources, such as dandelion root polysaccharides mainly made up of rhamnose, galactose, and arabinose (8) and *Pueraria lobata* root polysaccharides mainly consisted of glucopyranose (9), ginseng polysaccharides are mainly composed of acidic polysaccharides containing pectin and neutral polysaccharides including amyloid mixtures, dextrans, and arabinogalactans. Its bioactivities are closely related to the structure; for example, the strong antioxidant is attributed to its high content of uronic acid (10), low molecular weight (11), and a high degree of branching (12). Ginseng residue is a by-product of various commercial ginseng saponins (i.e., ginsenosides) extracts, and the residue yield after extraction has been increasing in recent years with the popularity of tonic foods and drinks. It is estimated that up to tens of thousand tons of ginseng residues are annually generated worldwide (13). More than this, most polysaccharides possessed by the ginseng root remain in the extraction residue that cannot be fully recovered and utilized, leading to resource waste and environmental pollution, especially water and soil pollution. Hsu et al. (14) analyzed the chemical structures and contents of ginsenosides in the extraction residue of the American ginseng root, and Hua et al. (15) studied the function of soluble dietary fiber from ginseng residue. However, there are few studies on coating fruits with polysaccharides extracted from plants, especially ginseng residues, to prolong their shelf-life.

Strawberry is one of the most fragile, perishable, and vulnerable fruits to mechanical damage, wilting, and physiological degradation, making its preservation a thorny problem. Similarly, the market for fresh-cut fruits such as fresh-cut apples has grown rapidly due to the increasing demand of consumers for high nutrition, freshness, and convenience (16). Deterioration and/or browning of these fruits, however, have become the main factors affecting their sensory and nutritional qualities (17). Several studies on fruit preservation using heat, ultrasound, and ascorbic acid treatments found that those methods did little to maintain their nutrition and commercial values (18). In this context, whether to find green strategies to maintain the nutrients and other quality indicators of vegetables and fruits has become a problem with both theoretical and commercial values. Such techniques should be sufficiently simple, and inexpensive and, when applied to production, should not adversely affect the quality but should even have a beneficial effect.

As a traditional polysaccharide extraction approach, the hot water extraction method can promote polysaccharides dissolution and diffusion through thermal effects. However, this method is time-consuming with limited extraction efficiency (19). The EDTA extraction method is also used to extract polysaccharides, especially pectin polysaccharides. However, a report by Zheng et al. (20) proved that the EDTA method has a high extraction efficiency for pectin-type polysaccharides but a low extraction efficiency for non-pectin-type polysaccharides. Recently, ultrasonic-assisted hot water extraction has been widely used and has proven to be an effective method for polysaccharides extraction, in that it can promote the dissolution of polysaccharides to a large extent through the double effect of increasing temperature and destroying cell walls by mechanical degradation (21–23). In this study, therefore, polysaccharides retained in extraction residue of ginseng root saponins, namely, ginsenosides-extracting residue polysaccharides (GRP), were recovered using the ultrasonic-assisted hot water extraction approach and their antioxidant activities were determined. GRP was then purified, and its structure and composition and those of its two purified fractions, namely GRP-1 and GRP-2, were characterized. Subsequent comprehensive evaluations of the effects of GRP-based coatings indicate that it would be a promising approach to maintaining the freshness and nutrition of fruits that are prone to decay and spoilage. This study is the first to obtain polysaccharides from the extraction residues of plant natural products and make them into a coating for the preservation of fresh food including fruits and vegetables. The study provides a new idea for further resource utilization of waste as well as the development of a new method to preserve food using polysaccharides or other natural bioactive compounds.

## Materials and methods

### Plant materials and reagents

Roots of 5-year-old ginseng (*Panax ginseng* C. A. Meyer) were purchased during the harvest season of field ginseng from Dandong, Liaoning province, China, and then air-dried overnight at 55 °C in an oven. The dried ginseng root was milled into powder by a grinder (FW135, Taist Instrument Co., Ltd., Tianjin, China) at 24,000 rpm, and passed through the 80-mesh sieve. Then the ginseng root powder was ultrasonically extracted (Power 500W, frequency 40 kHz; KQ-300DE NC ultrasonic cleaner, Kunshan ultrasonic instrument Co., Ltd., Jiangsu, China) with 70% aqueous ethanol at 30 °C for 30 min. The yield of ginsenosides extract from the supernatant accounted for 26.44% of the ginseng root at a dry weight base, and the precipitate (residue) was used as a raw material for the extraction of GRP.

Strawberry (*Fragaria ananassa* Duch.) was harvested in March 2021 and bought from a local farmer. “Fuji” apple (*Malus domestica* Mill) was harvested in November 2020, immediately refrigerated at 4 °C, and purchased from a supermarket (MerryMart, Beijing, China) on the same day performing the coating experiments.

Reagents for antioxidant assays, including 2,2-diphenyl-1-picrylhydrazyl (DPPH•), 2,2'-azino-bis (3-ethyl-benzothiazoline)-6-sulphonic acid (ABTS•^+^), were purchased from Sigma Chemical Co. (St. Louis, MO, United States). Folin phenol was purchased from the Tianli Chemical Reagent Co. (Tianjin, China). The DEAE-52 cellulose and diagnostic kits for assaying activities of polyphenol oxidase and catalase and the contents of ascorbic acid and malondialdehyde were purchased from Beijing Solarbio Science and Technology Co., Ltd. (Beijing China). Other reagents of analytical grade were bought from Sinopharm Chemical Reagent Co. Ltd. (Beijing, China). Ultra-pure water was prepared using a Milli-Q50 SP Reagent Water System (Millipore Corporation, Billerica, MA, United States).

### Extraction and purification of GRP and its fractionation into GRP-1 and GRP-2

The ginseng residue after extraction of ginsenosides was further extracted three times for obtaining GRP using distilled water (1:15, g/ml) at 80 °C for 30 min with an ultrasonic cleaner (KQ-300DE, Kunshan Ultrasonic Instrument Co., Ltd, Jiangsu, China). After centrifugation at 4,000 rpm and 4 °C for 10 min, the three supernatants were combined and concentrated at 55 °C in a rotary evaporator (RE-52A, Shanghai Yarong Biochemical Instrument Factory, Shanghai, China) under reduced pressure, and a four-fold volume of 95% ethanol was added to the concentrate, which was then kept at 4 °C overnight to precipitate the polysaccharides.

The precipitate was collected and lyophilized with a freeze dryer (FreeZone, Labconco Corporation, Kansas City, MO, United States) under a vacuum pressure of 10 Pa at −50 °C to obtain dried GRP for subsequent experiments. The GRP obtained by this method was water-soluble polysaccharides at room temperature and was purified and separated with an ion-exchange column chromatography of DEAE-52 cellulose, which has increasingly attracted attention as the stationary phase due to its stable physical and chemical properties, high separation efficiency, and short equilibrium and elution time. Polysaccharides are adsorbed on the cellulose depending on Coulomb force and charge difference. Then, the GRP of different fractions, such as neutral and acidic polysaccharides, can be eluted by using different concentrations of sodium chloride solution. Therefore, this method was chosen to separate neutral polysaccharides and acidic polysaccharides. In addition, it can also remove some pigments (24–27). GRP thus purified with its ratio to cellulose of 500 mg to 50 g was eluted with a step-wise gradient of 0 and 0.1 mol/L NaCl aqueous solution at atmospheric pressure and a flow rate of 1 ml/min controlled by a constant-flow pump (BT-100, Shanghai Huxi Analysis Instrument Factory Co., Ltd. Shanghai, China). The eluants were collected in 5 ml/tube and the total sugar content of the GRP and eluant in each tube was monitored using the phenol-sulfuric acid method with glucose as the standard (28). Then, the main two fractions were collected, dialyzed, and lyophilized to obtain the purified fractions, namely GPR-1 and GRP-2. The protein of GRP was measured using bovine serum albumin as the standard as reported by Bradford (29).

### Determinations of molecular weight and monosaccharides composition of GRP, GRP-1, and GRP-2

Molecular weights of the samples were measured by gel permeation chromatography (GPC) (Waters 1525, Waters Technology Co., Ltd, Milford, MA, United States) equipped with a PL aquagel-OH MIXED column (300 mm × 7.5 mm; particle size 8 μm; Agilent Technology Co., Ltd, Palo Alto, CA, United States) and a Waters 2414 refractive index detector (Waters Technology Co., Ltd, Milford, MA, United States) at 30 °C. The column was eluted with distilled water at a flow rate of 0.5 ml/min. The calibration curve was performed using dextran standards with different molecular weights (30).

For monosaccharides composition of polysaccharides, 10 mg samples were hydrolyzed using 2 ml of 2 M Trifluoroacetic acid at 110 °C for 8 h in an oven. The hydrolyzed products were evaporated to dryness and derivatized using the following method. Sodium hydroxide at 0.6 M of 250 μl and PMP (1-phenyl-3-methyl-5-pyrazolone) at 0.4 M of 500 μl was added to the 250 μl hydrolyzed sample. The mixture was placed in the water bath at 70 °C and incubated for 1 h. After cooling to room temperature, hydrochloric acid at 0.3 M of 500 μl and chloroform of 1 ml were added. Then the reaction system was centrifuged at 4,000 g for 10 min. The supernatant was analyzed by high-performance liquid chromatography (HPLC) on an Ultimate 3000 Series HPLC system coupled with a UV-detector (250 nm; Thermo Fisher Scientific Co., Ltd, Waltham, MA, United States) and equipped with an Xtimate-C18 column (4.6 mm × 200 mm, 5 μm; Welch Technology Co., Ltd, Shanghai, China) at 30 °C. The mobile phase was a mixture of phosphate-buffered saline (0.05 M, pH 6.7) and acetonitrile (83:17, v/v). The flow rate was set at 1.0 ml/min. The monosaccharides standards were derived and analyzed in the same manner (31).

### FT-IR and NMR spectra of GRP, GRP-1, and GRP-2

FT-IR spectra of the polysaccharides were recorded using the KBr-disk method with a Fourier transformed infrared spectrophotometer (Perkin Elmer Frontier, PerkinElmer Management Co., Ltd, Waltham, MA, United States). Briefly, the samples were ground with KBr powder and then pressed into a 1-mm pellet for FT-IR spectral analysis within the scanning range of 4,000–600 cm^−1^.

NMR spectra of the polysaccharides were carried out on an NMR spectrum (Bruker Avanchiiihd 500, Bruker Technology Co., Ltd, Billerica, MA, United States) at 30 °C. Dried polysaccharides of 50 mg were dissolved completely in 1 ml deuterium water or deuterium oxide (D_2_O; 99.97%) and then lyophilized. This procedure was repeated twice more, and the final sample was once again dissolved in 0.5 ml of D_2_O (99.97%). After the treatment, the ^1^H-NMR and the ^13^C-NMR spectra were measured.

### GRP coatings of strawberry and fresh-cut apple

Strawberries with uniform size (roughly 5.0 × 2.5 × 2.5 cm^3^) and ripeness and without visual defects, were cleaned with deionized water and divided into three groups: control group, lower concentration GRP-coating group (L-GRP), and higher concentration GRP-coating group (H-GRP). Subsequently, they were dipped for 5 min in deionized water and 5 and 20 mg/ml GRP deionized water solutions, respectively. Then they were placed into sterile polypropylene trays which were covered with a lid after the fruits were air-dried, and incubated for 96 h in a controlled growth chamber (Life Technology Co., Ltd, Ningbo, China) set at a temperature of 20 ± 1 °C and relative humidity of 25 ± 2 %. The experiments of the three treatment groups were completed in the same “96 h,” and each treatment group was repeated three times in three consecutive “96 h.”

Apples were rinsed gently with deionized water by hands, air-dried, cored, and cut into cubes at a size of 3 × 3 × 3 cm^3^ with a sanitized sharp stainless-steel knife. All fresh-cut apple cubes were also divided into three groups (i.e., control, L-GRP, and H-GRP) and subjected to the same treatments as described above for strawberries, including the repetition of the experiment. Considering that the shelf-life of fresh-cut fruit was shorter than that of non-fresh-cut fruit, a period of 48 h, instead of 96 h, was selected to incubate the fresh-cut apples.

### Determinations of weight loss, firmness, soluble solids, and titratable acidity of fruits

Initial weight at 0 h was used to calculate the weight loss rate of strawberry and fresh-cut apple. Six strawberries or fresh cuts of apple in each group were weighed. The weight loss rate was calculated according to the following formula:


(1) $$\begin{array}{c} \text{Weight loss rate }(\%)=(W0\;-\;W1)/W0\;\times \;100 \end{array}$$

where *W0* is the weight of samples at 0 h and *W1* is that of stored samples at the corresponding hours.

The firmness of strawberry and fresh-cut apple was measured by a fruit hardness tester (GY-5A, Aipu Measuring Instruments Co., Ltd, Hengzhou, China) using a flat 11-mm cylindrical probe, which was pressed into three different points in the central zone of each fruit for a depth of 10 mm. Soluble solids in strawberry and fresh-cut apple pulp were determined by an Abbe Refractometer (2WAJ, Shanghai Optical Instrument Co., Ltd, Shanghai, China) and expressed as percentages.

For titratable acidity analysis, 5 g strawberry or fresh-cut apple pulp was homogenized and placed into a 50-ml volumetric flask and diluted to scale with distilled water. The flask was left to stand for 30 min before filtration, and 20 ml filtrate was then titrated to pH = 7 with 0.1 mol/L NaOH (32). The total titratable acidity of the diluted pulp solution was calculated using the following formula:


(2) $$\begin{array}{l} TA\;\left(\%\right)=[(VNaOH\;\times \text{ M }\times \;0.064\;\times \;V0)/(V1\;\times \;m)]\\ \;\times \;100 \end{array}$$

where *VNaOH* is the volume of NaOH used for titration in ml, M is the molarity of the NaOH solution, the value of 0.064 is the conversion factor for citric acid, *V0* is the volume of the volumetric flask, *V1* is the filtrate volume, and *m* is the mass of the pulp.

### Determinations of relative electrolyte leakage and malondialdehyde of fruits

Relative electrolyte leakage was evaluated as reported by Ali et al. (33). Briefly, 2 g pulp was incubated in 100 ml distilled water at 25 °C for 1 h and then the initial electrolyte leakage (*R0*) was measured with the conductivity meter (LICHEN CT-20, Guangzhou China). The same solution in a wide-mouth bottle with a lid was heated in a boiling bath for 1 h and the final electrolyte leakage (*R1*) was measured after cooling. Relative electrolyte leakage was calculated with the following formula:


(3) Relative electrolyte leakage (%) =R0/R1 × 100

Malondialdehyde was assayed with the thiobarbituric acid method according to the manufacturer's instructions of the commercial kit. Briefly, strawberry or fresh-cut apple pulp (0.1 g) was homogenized in 1 ml trichloroacetic acid and the homogenate was centrifuged at 8,000 g for 10 min. The supernatant obtained was used for assay of malondialdehyde, and the result was expressed as nmol/g on a fresh weight basis.

### Determinations of ascorbic acid and total phenols of fruits

Ascorbic acid was assayed with the 2, 6-dichlorophenol indophenol method according to the manufacturer's instructions of the commercial kit. Briefly, a 0.1-g sample of pulp was blended in 1 ml oxalate to extract ascorbic acid. The mixture was centrifuged at 8,000 g and 4 °C for 10 min, and then the supernatant was taken for ascorbic acid assay. Ascorbic acid was expressed as nmol/g on a fresh weight basis.

For measurement of total phenols, 2 g pulp was homogenized in 20 ml aqueous ethanol (80 %, v/v) and then extracted with an ultrasonic cleaner for 30 min. The mixture was centrifuged at 8,000 g for 10 min at room temperature, and the supernatant was used to measure the total phenols with the microplate reader (Bio-Rad xMark^TM^ Microplate Absorbance Spectrophotometer, Bio-Rad Technology Co., Ltd, MA, United States) according to the Folin-Ciocalteu colorimetric method (34). The content of total phenols was expressed as mg/g (relative to fresh weight).

### Assays of activities of polyphenol oxidases and catalase of fruits

Strawberry or fresh-cut apple pulp (0.1 g) was homogenized in 1 ml of a 0.1-M Na_2_HPO_4_-NaH_2_PO_4_ buffer (pH 7.0) and then centrifuged at 8,000 g for 10 min at room temperature. The supernatant was used for assays of the aforesaid polyphenol oxidases and catalase activities, which were assayed according to the manufacturer's instructions for the commercial kits. Polyphenol oxidase activity was assayed by monitoring absorbance change at 410 nm and is expressed as a 0.05 variation in the absorbance per minute per gram per milliliter sample solution. For the activity of catalase, H_2_O_2_ decomposition was measured at 240 nm. Catalase activity unit is defined as the ability of each gram of tissue to catalyze 1 μmol of hydrogen peroxide per minute in the reaction system.

### Evaluations of DPPH•, ABTS•^+^, and OH• antioxidant capacities of GRP and fruits

DPPH•, ABTS•^+^, and OH• scavenging assays, often used to determine the antioxidant activity of plant polysaccharides (e.g., 5, 8, 22) due to their relatively simple operation and high accuracy, were selected to analyze the antioxidant capacity of GRP. GRP was dissolved in distilled water at 0.5, 1, 2, 4, 6, 8, 10, 12, 14, 16, 18, 20, and 22 mg/ml for assaying antioxidant activities. A total of 2 g of strawberry or fresh-cut apple pulp was homogenized in 20 ml aqueous ethanol (80 %, v/v) and centrifuged at 8,000 g for 10 min. Then the supernatant, namely 80 % ethanol extract solution, was used to evaluate the antioxidant.

DPPH• scavenging activities of GRP and fruits and ABTS•^+^ scavenging activity of GRP was determined using ascorbic acid as the standard as reported by Li et al. (35) and calculated by the following formula:


(4) Scavenging activity (%)=(A0 − A1)/A0 × 100

where *A1* is the absorbance of each sample or ascorbic acid, and *A0* is that of a control prepared without adding the sample.

OH• scavenging activities of GRP, ethanol extract solution of strawberry or fresh-cut apple pulp or ascorbic acid was determined as reported by Liu et al. (36). The inhibition rate was calculated by the following formula:


(5) Inhibition rate (%)=[1 − (A0 − A1)/A2] × 100

where *A0* is the absorbance of the reaction systems with various concentrations of polysaccharides, *A1* is that when salicylic acid was replaced with distilled water, and *A2* is that of blank control in which the polysaccharides were replaced by distilled water.

### Statistical analysis

Three parallel experiments were performed for each treatment in this study, and data were shown as means ± standard deviations of a biological triplicate. The statistical significance of one-way analysis of variance (ANOVA) was tested using SPSS software (ver. 20.0; SPSS Inc., Chicago, IL, United States). Different letters indicate significance at *p* < 0.05 based on the Tukey test. Principle component analysis (PCA) was operated by the Origin 2021 software (OriginLab Massachusetts, United States).

## Results and discussion

### Characterization of GRP

#### Chemical compositions of GRP, GRP-1, and GRP-2

**Figure 1:**
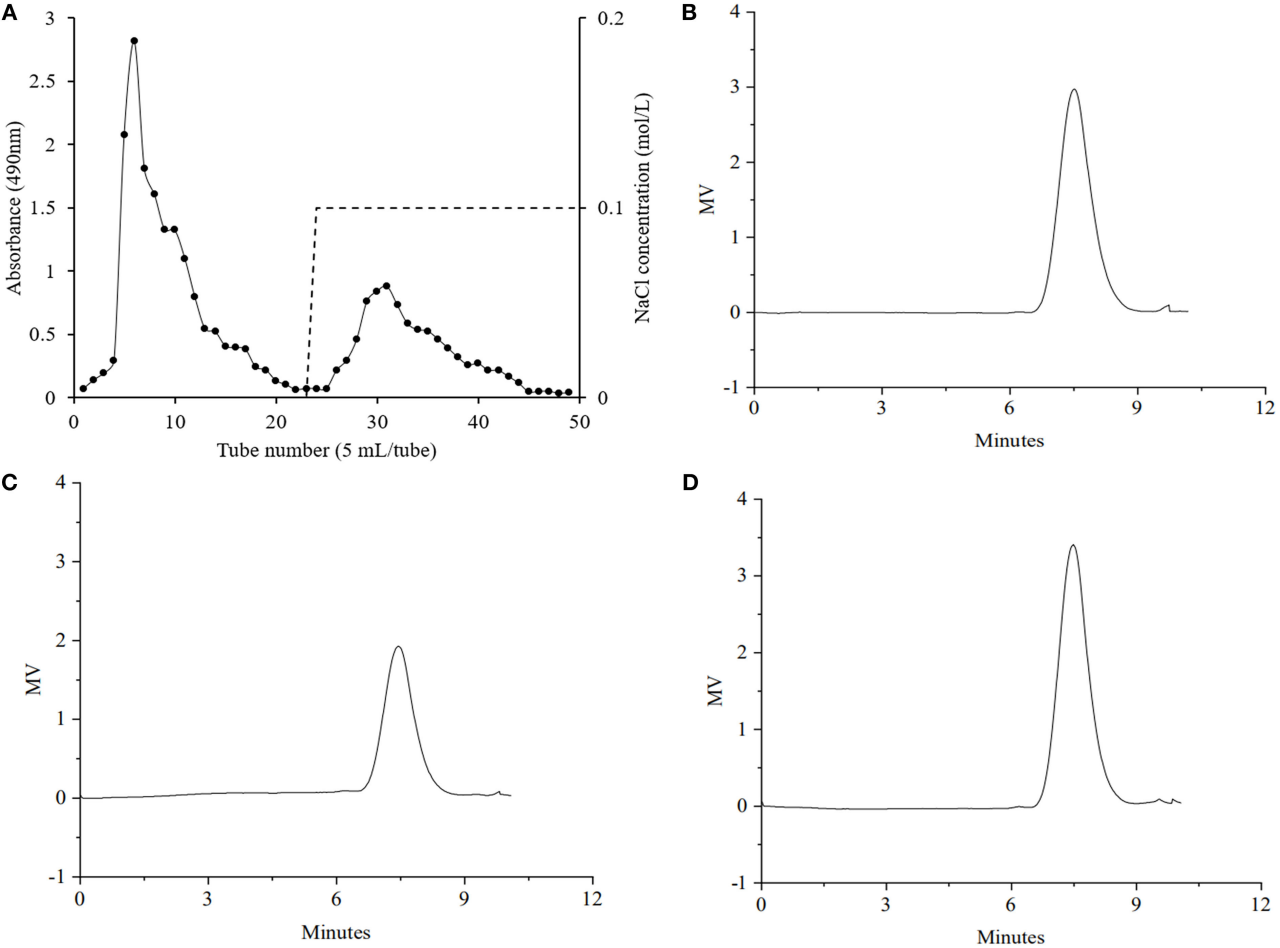
Elution curve of GRP using DEAE-52 by gradient concentrations of NaCl **(A)** and gel permeation chromatography chromatograms of GRP **(B)**, GRP-1 **(C)** and GRP-2 **(D)** NaCl concentrations of 0 and 0.1 M were marked on the right-side Y axis. GRP, ginsenosides-extracting residue polysaccharides; GRP-1 and GRP-2, fractions separated by 0 M and 0.1 M NaCl from GRP, respectively.

The yield of GRP, which was extracted using hot water and then precipitated with ethanol from extraction residue of ginseng root saponins, was approximately 16.15%, and its relative content of total sugars was 61.56% (Table 1). Figure 1A shows that the elution curve obtained using anion-exchange chromatography presented two independent peaks, of which the peak on the left was GRP-1 with a yield of 3.25%, and that on the right was GRP-2 with a yield of 0.93%. Total sugars of GRP-1 and GRP-2 were 64.29% and 67.21%, respectively (Table 1). The yield of GRP was within the reasonable range. For example, the polysaccharides yields of *Dioscorea hemsleyi* (37), *Hovenia dulcis* (38), and dandelion root (8) are 3.82%, 8.6%, and 44.95%, respectively.

**Table 1:** Physicochemical properties of the polysaccharides isolated from ginseng residue.

**Fraction**	**GRP**	**GRP-1**	**GRP-2**
Yield^*^ (%)	16.15 ± 2.21^a^	3.25 ± 0.85^b^	0.93 ± 0.01^c^
Total Surgurs (%)	61.56 ± 2.47^bc^	64.29 ± 1.05^b^	67.21 ± 2.42^a^
Proteins (%)	0.19 ± 0.06	-^***^	-
Mn (KDa)^**^	106.28^c^	117.34^a^	115.69^b^
Mw (KDa)	171.95^a^	190.99^a^	182.7^a^
Monosaccharide composition (mol%)			
Glucose	94.47 ± 0.32^b^	97.55 ± 0.12^a^	78.05 ± 0.10^c^
Galactose	1.49 ± 0.20^b^	0.49 ± 0.01^c^	12.20 ± 0.05^a^
Arabinose	0.54 ± 0.10^b^	0.24 ± 0.00^c^	5.13 ± 0.01^a^
Mannose	0.48 ± 0.01^b^	0.27 ± 0.03^c^	1.82 ± 0.01^a^
Rhamnose	0.14 ± 0.05^b^	0.04 ± 0.00^c^	1.19 ± 0.08^a^
Ribose	0.22 ± 0.02^b^	0.27 ± 0.01^b^	0.66 ± 0.02^a^
Glucuronic acid	1.38 ± 0.26^a^	0.81 ± 0.09^b^	0.40 ± 0.00^c^
Xylose	0.41 ± 0.11^a^	0.23 ± 0.15^a^	0.38 ± 0.02^a^
Fucose	0.53 ± 0.19^a^	0.07 ± 0.26^b^	0.14 ± 0.00^b^
Galacturonic acid	0.1 ± 0.01^a^	0.05 ± 0.01^b^	0.03 ± 0.00^b^

* Percentages of the absolute weight of each index over dry weight of the extraction residue of ginseng saponins used for recovery of polysaccharides;

** Mn, number-average molecular weight; Mw, weight-average molecular weight. Results were presented as mean ± SD of three independent experiments (n = 3). Different letters indicate significant differences at p < 0.05 based on Tukey test.

*** means not detected.

It is worth noting that the relative content of proteins of the GRP was merely 0.19%, making the deproteinization unnecessary, and thus, was omitted in this study. According to literature (39–41), the rest of each extract may be mono- or oligo-saccharide molecules, ash and/or some uronic acids. Since this study mainly aimed to explore whether the structure of GRP was destroyed, further purification or clarification of components other than GRP was not performed. In addition, compared with the polysaccharides and fractions extracted directly from the ginseng root by Kim et al. (40), this study increased (*p* < 0.05) the sugar content of GRP-2.

The molecular weight is a major parameter influencing the bioactivity of polysaccharides. In principle, the solubility of the polysaccharides increased with the decrease of molecular weight, and the polysaccharides with high solubility had stronger antioxidant activity (42). It can be seen from the chromatograms that GRP, GRP-1, and GRP-2 possessed symmetric single peaks (Figures 1B–D), and thus could be considered uniform polysaccharides. As shown in Table 1, the number-average molecular weights (Mn) and weight-average molecular weights (Mw) of GRP, GRP-1, and GRP-2 were 106.28 and 171.95, 117.34 and 190.99, and 115.69 and 182.70 KDa, respectively. The Mn of either GRP-1 or GRP-2 was higher (*p* < 0.05) than that of GRP, however, their Mw showed no difference (*p* > 0.05). The molecular weight of GRP in this study was like that of water-soluble polysaccharides from ginseng root reported by Zhang et al. (43), which was approximately 150 KDa.

The monosaccharides composition of GRP, GRP-1, and GRP-2 was analyzed by HPLC (Table 1). Although their ten monosaccharides were the same in species, the molar percentage of each monosaccharide in GRP, GRP-1, and GRP-2 and the order from high to low were different. For details, first, glucose accounted for the majority of GRP (94.47%), GRP-1 (97.55%), and GRP-2 (78.05%) and they differed from each other (*p* < 0.05). Second, the molar percentages of galactose, arabinose, and mannose as well as rhamnose in GRP-2 accounted for more than 10%, 5%, and 1%, respectively, and were higher (*p* < 0.05) than those of GRP and GRP-1, indicating that it is a mixture of heteropolysaccharides. Our results of GRP-1 were consistent with those reported by Kim et al. (40) and Zhang et al. (43). However, the glucose of GRP-2 was higher and its galactose, arabinose, and galacturonic acid were lower in relative contents than their reported results. These might be caused by the loss of specific monosaccharides during ginsenosides extraction, such as partial loss of galactose, arabinose, and galacturonic acid as pectin compositions, leading to an increase in the proportion of glucose in GRP.

#### Structural properties of GRP, GRP-1, and GRP-2

**Figure 2:**
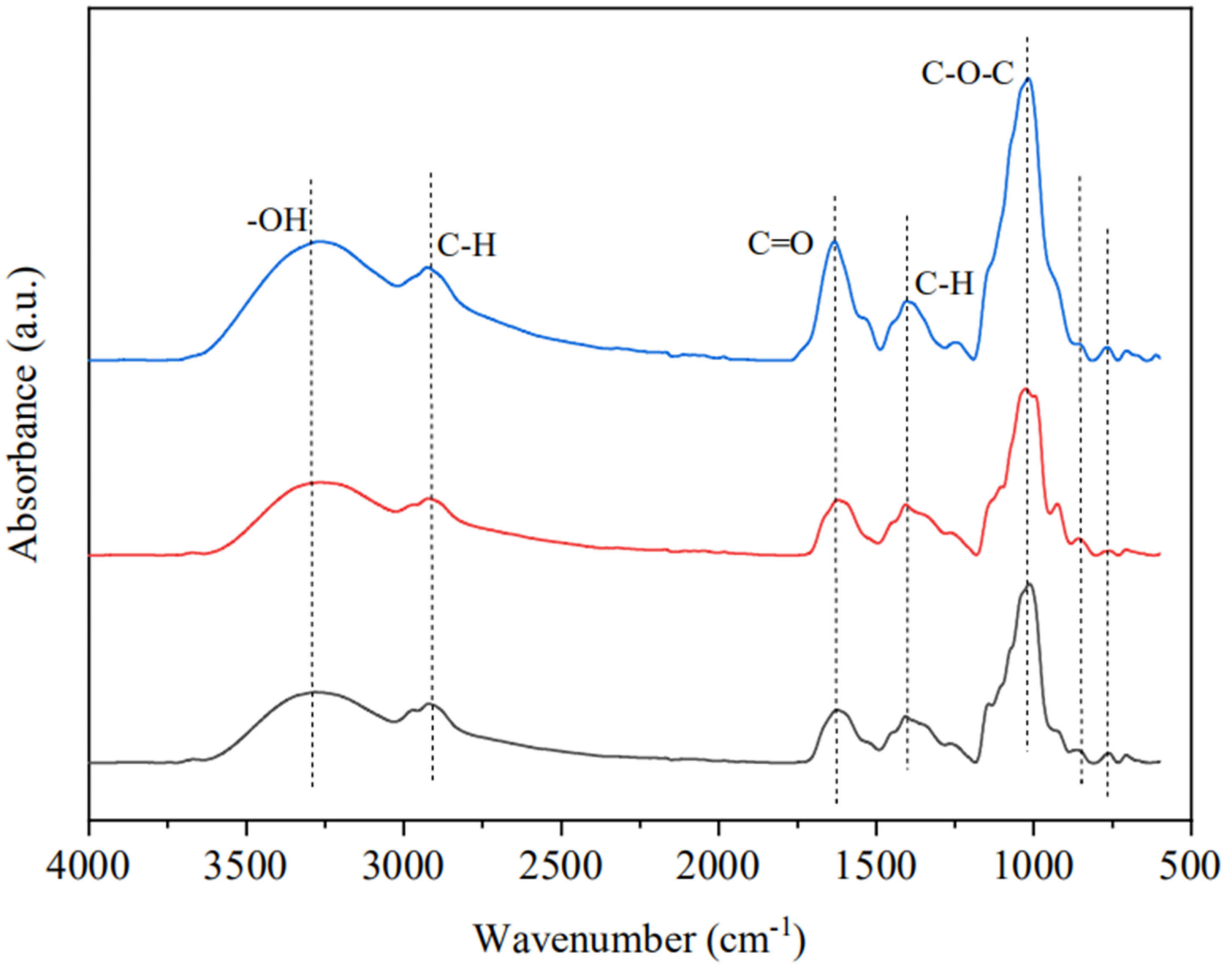
Fourier transform infrared spectroscopy of GRP, GRP-1, and GRP-2. The black, red and blue curves represent the spectra of GRP, GRP-1, and GRP-2, respectively. GRP, ginsenosides-extracting residue polysaccharides; GRP-1 and GRP-2, fractions separated by 0 M and 0.1 M NaCl from GRP, respectively.

To understand the structural differences between GRP, GRP-1, and GRP-2, we carried out the FT-IR and NMR assays. FT-IR spectra of GRP, GRP-1, and GRP-2 (Figure 2) had a broad characteristic absorption peak, which extended from 3,320 to 3,250 cm^−1^, and a weak peak at around 2,915 cm^−1^. These two peaks were attributed to the stretching vibration of -OH and C-H, while C-H also led to a weak absorption at approximately 1,396 cm^−1^ due to deformation vibration (44). While the absorbance in the range of 1,000-1,100 cm^−1^ could be assigned to C-O-C, which suggested that sugar rings were pyranose rings (45, 46), a small scale of absorption peak around 1,634 cm^−1^ suggested the existence of C=O. On the other hand, in the spectrum of GRP and that of GRP-1 and GRP-2, the peaks close to 848 cm^−1^ suggested the existence of α-D-glucopyranose (47), whereas the absorbance of GRP-2 in 1,634 cm^−1^ and 1,000-1,100 cm-1 were stronger than those of GRP and GRP-1, indicating its existence of more C=O and C-O-C groups, which may be related to their differences in monosaccharide composition (Table 1).

^1^H and ^13^C NMR spectra of GRP, GRP-1, and GRP-2 at 500-MHz were also measured (Supplementary Figure S1). The ^1^H NMR spectra (Supplementary Figures S1A–C) were mostly crowded in a region ranging from δ3 to δ5 ppm which was typical of polysaccharides (48). The ^1^H signal at δ4.70 ppm was due to heavy water. The chemical shifts from δ3.5 to δ4.5 ppm were assigned to the protons of carbons C-2 to C-6 of the glycosidic ring (49) in GRP, GRP-1, and GPR-2. The shift information of carbon nuclei based on NMR spectra (Supplementary Figures S1D-F) is shown in Table 2. The results showed that the 4 possible types of configurations that existed in GRP, GRP-1, and GRP-2 included 1,4-α-Glcp, β-Galp, α-Araf, and 1,4-α-GalA. In addition, the corresponding carbon signals obtained from the ^13^C NMR spectra of GRP-2 (Supplementary Figure S1E) were assigned to δ97.67 ppm for C-1 of 1,2-α-Rhamnose (50, 51).

**Table 2:** The signals of chemical shifts in ^13^C NMR spectra of GRP, GRP-1 and GRP-2.

		**Chemical shifts (ppm)**					
		**C-1**	**C-2**	**C-3**	**C-4**	**C-5**	**C-6**
GRP^*^	1,4-α-Glcp^**^	-^***^	71.63	73.06	77.08	70.15	60.43
	β-Galp	103.63	81.32	72.64	74.52	68.45	60.05
	α-Araf	-	81.32	76.34	60.43	-	-
	1,4-α-GalA	-	67.5	70.14	81.32	71.41	-
GRP-1	1,4-α-Glcp	99.65	71.58	73.06	77.08	70.15	60.44
	β-Galp	-	-	72.71	74.41	-	60.64
	α-Araf	-	-	76.63	60.64	-	-
	1,4-α-GalA	-	67.52	70.15	80.64	71.45	-
GRP-2	1,4-α-Glcp	-	71.36	73.36	-	70.14	60.44
	β-Galp	-	81.3	83.81	73.36	68.41	-
	α-Araf	109.21	81.3	76.51	60.44	-	-
	1,4-α-GalA	-	68.41	70.14	-	71.36	-

* *GRP, ginsenosides-extracting residue polysaccharides; GRP-1 and GRP-2, fractions separated by 0 M and 0.1 M NaCl from GRP, respectively*;

** *Glcp, glucopyranose; Galp, galactopyranose; Araf, arabinofuranose; GalA, galactopyranouronic acid*.

*** *‘-' means not detected*.

The above results of monosaccharide composition, FT-IR, and NMR of GRP, GRP-1, and GRP-2 demonstrated that GRP contained mainly GRP-1 composed of starch-like glucans and a small portion of GRP-2 composed of starch-like glucans, rhamnogalacturonan-I (RG-I) pectin, and arabinogalactans which is one of the partial structures of most common pectin. These results were consistent with those of Kim et al. (40) and Zhang et al. (43). Meanwhile, it can be concluded that the isolated GRP was starch-like glucan rather than impurity starch due to the different extraction processes of polysaccharides and starch (52). However, the contents of RG-I, pectin, and arabinogalactan in this study were slightly low compared to those of the previous reports, which may be due to GRP-2 still containing a certain number of starch-like glucans. The best approach would be to continue to purify GRP-2 by column chromatography. However, the proportion of GRP-2 in GRP was very low, and the purpose of this study was to verify whether the extraction of ginsenosides in a previous stage would affect the structure of polysaccharides from ginseng residue, so further purification was not carried out. To verify whether the biological activities of GRP, such as antioxidant activity, were affected, the following experiments were performed.

#### Antioxidant capacities of GRP

**Figure 3:**
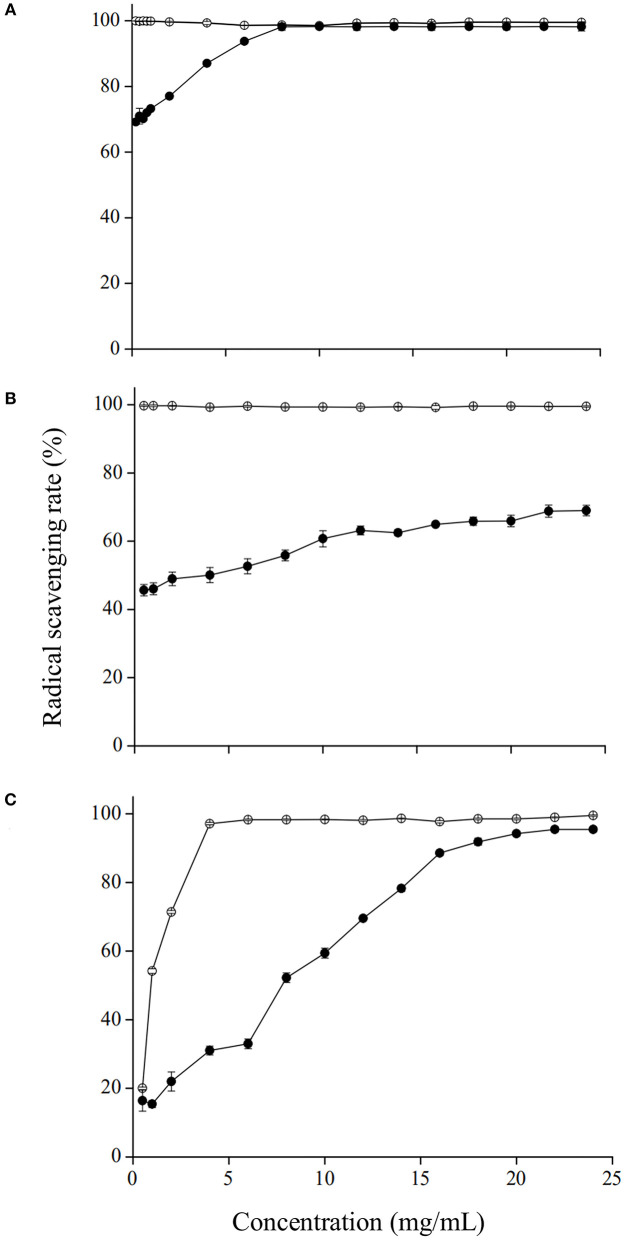
Antioxidant capacities of GRP to DPPH•**(A)**, ABTS•^+^ **(B)**, and OH• **(C)**. Ascorbic acid (open circles) was used as the standard for all the three assays. Data were calculated as the radical scavenging rate (%). Results were presented as mean ± SD of three independent experiments (*n* = 3). GRP, ginsenosides-extracting residue polysaccharides.

Antioxidant properties of the GRP were subsequently investigated by DPPH•, ABTS•^+^, and OH• scavenging assays. As shown in Figure 3, ascorbic acid, used as the positive control, showed substantial scavenging activities even at relatively low concentrations, affirming the results reported by Kim et al. (40). DPPH•, ABTS•^+^ , and OH• scavenging activities of GRP all increased in a concentration-dependent manner. At the concentrations of 8 mg/ml (Figure 3A) and 22 mg/ml (Figure 3C), its DPPH• and OH• scavenging rates were 98.22 % and 95.49 %, respectively, which exhibited no differences (*p* > 0.05) to the corresponding positive controls. The scavenging rate of GRP for ABTS•^+^ at the concentration of 22 mg/ml was 69.00 % (Figure 3B), lower (*p* < 0.05) than those for DPPH• and OH• as well as the positive control. The reason for this difference may be that a certain amount of ABTS•^+^ coexisting with K_2_S_2_O_8_ is not easy to separate, which interferes with the results. Moreover, slope rates of the concentration-dependent curves were different, with those for ABTS•^+^ and DPPH• being the lowest and highest, respectively. The scavenging capacities of the present study are stronger than those reported previously with polysaccharides of Chinese water chestnut peels (53) and *Notopterygium franchetii* Boiss (54). The GRP in this study still had strong antioxidant activity, which was probably related to the relatively higher levels of glucuronic acid and galacturonic acid maintained despite the ginsenosides-extraction process, while those of both the two uronic acids were significantly reduced (*p* < 0.05) in its two fractions GRP-1 and GRP-2. The presence and content of uronic acids are important indexes to reflect the antioxidant activity of polysaccharides, and the contribution of glucuronic acid is greater than that of galacturonic acid (55). Given the above characteristics, GRP rather than its two fractions could be used as a good antioxidant in the storage and transportation of fruit and vegetables with high moisture.

Ginseng is a beneficial herbal medicine for health, and the GRP was extracted only using deionized water and ethanol without toxic reagents. In addition, most of the literatures on polysaccharides obtained from plants have not conducted cytotoxic test [e.g., (53, 54)], so this study also did not conduct this test. Anyway, for the sake of security, we will study the cytotoxic test of GRP in further study. We have also determined the antibacterial activity of GRP with different concentrations (5, 10, and 20 mg/ml) against *Escherichia coli* ATCC 25922 and *Staphylococcus aureus* ATCC 25923 by using the Oxford cup method. By observation, we found that there was no bacteriostatic circle in each group, indicating that GRP had no significant antibacterial activity (data not shown). Although antibacterial substances such as plant essential oils can be added to the GRP coating to enhance the antibacterial activity of the coating, the structure and bioactivity of GRP may be destroyed. In view of this, this study focused on the effects of GRP as an antioxidant on strawberries and fresh-cut apple preservation. Following this idea, we carried out the following research.

### Effects of GRP-coating on the preservation of strawberry and fresh-cut apple

#### Effects of GRP-coating on six quality parameters

**Figure 4:**
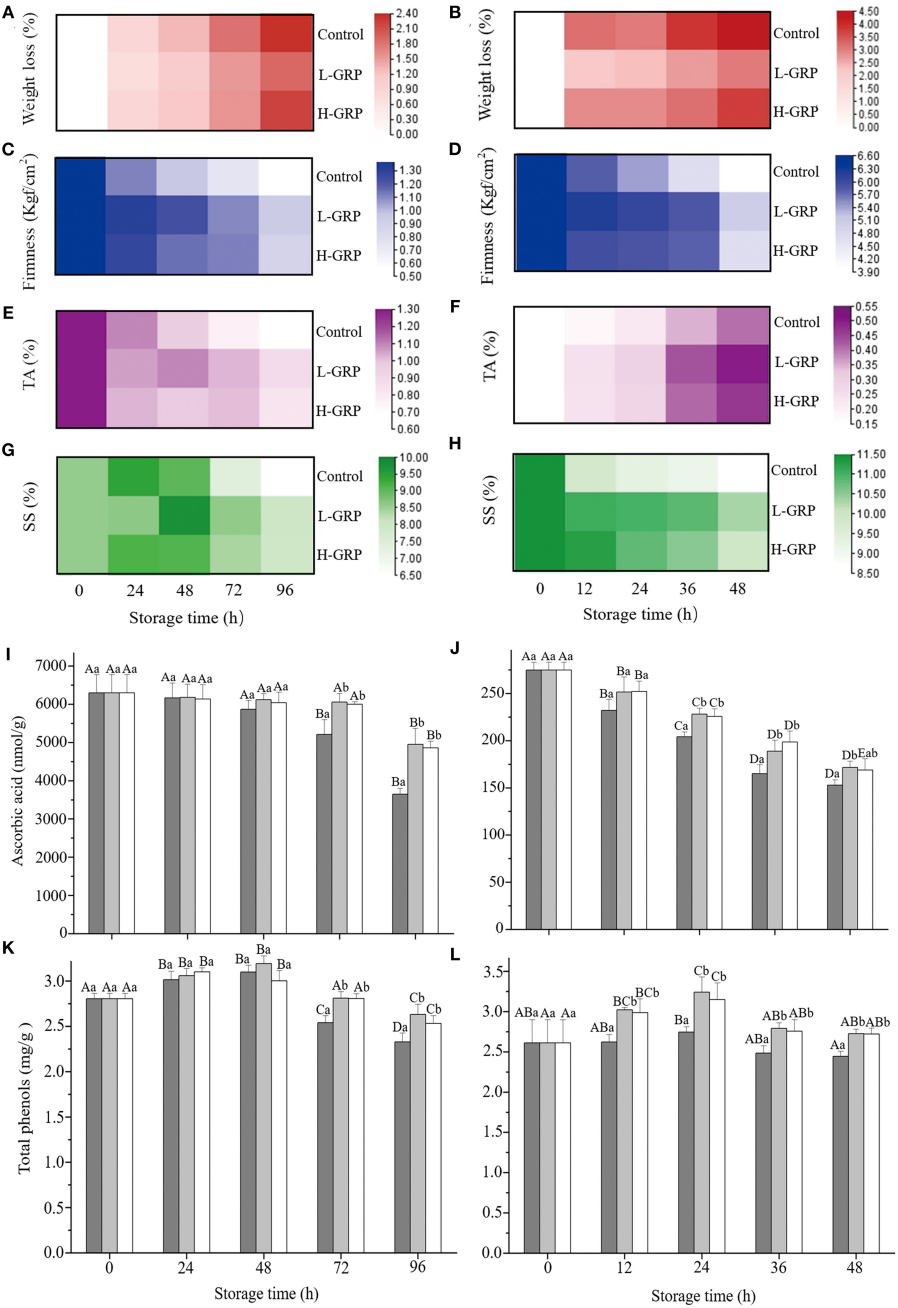
Changes of weight loss **(A,B)**, firmness **(C,D)**, titratable acidity **(E,F)**, soluble solids **(G,H)**, ascorbic acid **(I,J)**, and total phenols **(K,L)** in strawberry **(A,C,E,G,I,K)** and fresh-cut apple **(B,D,F,H,J,L)** respectively, during storage at 20 ± 1 °C and a relative humidity of 25 ± 2 % after treatments with deionized water or GRP aqueous solutions. GPR, ginsenosides-extracting residue polysaccharides; Control (black columns), fruits treated with deionized water; L-GRP (gray columns) and H-GRP (white columns), fruits treated (coated) with GRP at 5 and 20 mg/ml in respective; TA, titratable acidity; and SS, soluble solids. The depth of the legend color represents the size of the corresponding value. Results were presented as mean ± SD of three independent experiments (*n* = 3). Different lower-case and upper-case letters indicate significant differences between and within groups, respectively, at *p* < 0.05 based on Tukey test.

Given the strong antioxidant activity of GRP, we next investigated their ability to extend the shelf-life of fruit. Weight loss, firmness, titratable acidity, and soluble solids are often used to evaluate the quality changes of fruit or vegetables with high moisture during storage and transportation (56). Both weight (Figures 4A,B) and firmness (Figures 4C,D) of all the three samples (i.e., control, L-GRP, and H-GRP) showed similar trends of gradual decrease during the storage of the two fruits, whereas titratable acidities of the strawberry and fresh-cut apple showed opposite trends of changes (Figures 4E,F), which were possibly due to differences in their composition of organic acids. For the changes of soluble solids, it first increased then decreased in the strawberry (Figure 4G) and decreased in the fresh-cut apple (Figure 4H). In addition, compared with the two GRP-treated groups, soluble solids of the control of fresh-cut apple sharply declined at 12 h, which may be due to the apple being a climacteric fruit, thus in its aging process, the respiratory rate accelerates, speeding up the consumption of substrates. More importantly, GRP coatings on both the strawberry (Figures 4A,C,E,G) and fresh-cut apple (Figures 4B,D,F,H) improved (*p* < 0.05) all the four indexes for extending their shelf-life. The results were like the effects of chitosan-pectin coating on fresh-cut cantaloupe (4).

Ascorbic acid levels of the control of strawberry kept unchanged then decreased (*p* < 0.05) (Figure 4I), whereas those of the fresh-cut apple decreased (*p* < 0.05) and then kept unchanged (Figure 4J). Nevertheless, compared to their controls, L-GRP and H-GRP coatings on strawberries and fresh-cut apples reduced the decline of their ascorbic acid levels from 72 and 12 h, respectively (Figures 4I,J). Total phenols of the control of strawberry first increased then decreased (*p* < 0.05) (Figure 4K), while those of the fresh-cut apple kept unchanged except that at the 24 h which were higher (*p* < 0.05) than that at the 48 h (Figure 4L). Both the L-GRP and H-GRP coatings to strawberries and fresh-cut apples maintained their total phenols at higher levels also from 72 and 12 h, respectively (Figures 4K,L). This finding was like the reported finding that Alginate oligosaccharide “elevated” the level of total phenols in strawberries (57).

#### Effects of GRP coating on five antioxidant parameters

**Figure 5:**
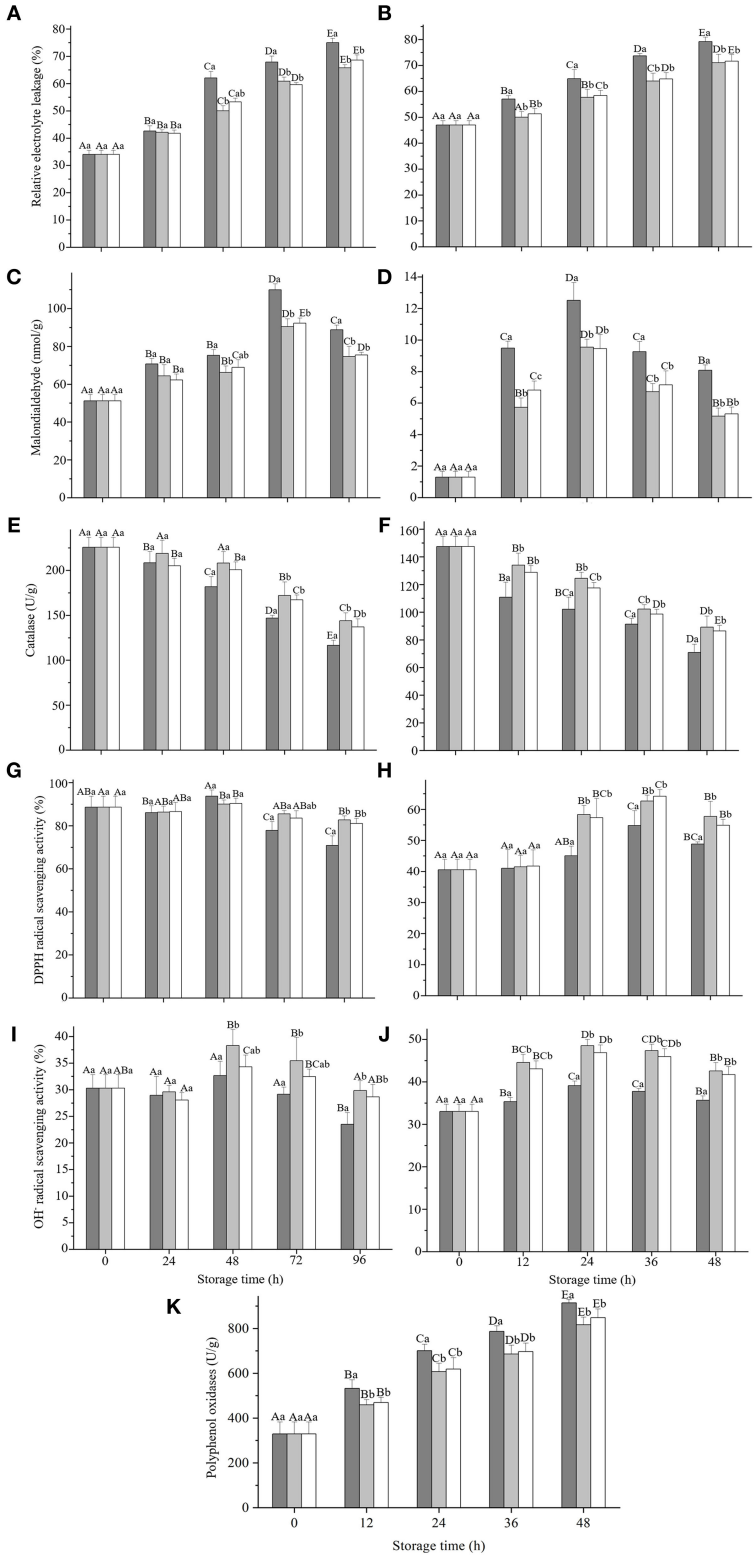
Changes of relative electrolyte leakage **(A,B)**, malondialdehyde formation **(C,D)**, catalase activity **(E,F)**, DPPH• scavenging activities **(G,H)**, and OH• scavenging activities **(I,J)** in strawberry **(A,C,E,G,I)** and fresh-cut apple **(B,D,F,H,J)** respectively, and polyphenol oxidases activity of fresh-cut apple **(K)** during storage at 20 ± 1 °C and a relative humidity of 25 ± 2 % after treatments with deionized water or GRP aqueous solutions. GPR, ginsenosides-extracting residue polysaccharides; Control (black columns), fruits treated with deionized water; L-GRP (gray columns) and H-GRP (white columns), fruits coated with GRP at 5 and 20 mg/ml in respective. Results were presented as mean ± SD of three independent experiments (*n* = 3). Different lower-case and upper-case letters indicate significant differences between and within groups, respectively, at *p* < 0.05 based on Tukey test.

To evaluate whether the GRP coating affected the antioxidant capacities of strawberries and fresh-cut apples during storage, we measured the parameters related to antioxidant activity. Relative electrolyte leakages of strawberry (Figure 5A) and fresh-cut apple (Figure 5B) steadily increased (*p* < 0.05), whereas the malondialdehydes of the two fruits (Figures 5C,D) increased (*p* < 0.05) only up to 72 and 24 h, respectively, then reduced (*p* < 0.05). Nevertheless, both L-GRP and H-GRP suppressed (*p* < 0.05) the relative electrolyte leakage and malondialdehyde formation compared to those of their corresponding controls, with no significant differences observed between L-GRP and H-GRP (Figures 5A–D). These suppressions by L-GRP and H-GRP coatings might have resulted from their prevention of the fruits from oxygenation, which might have been caused by their maintenance of the fruits' antioxidant capacities that were subsequently investigated. The results were like the effects of *Oudemansiella radicata* polysaccharides on the postharvest quality of oyster mushroom by Liu et al. (58). However, the value of MDA had been increasing in their study, which is slightly different from this study. A possible explanation for the decrease of MDA might be that the decline of cell metabolism capacity with the decay of fruit during storage resulted in a decrease in lipid peroxidation which is the main cause of MDA production.

As shown in Figures 5E,F, catalase activities in strawberry and fresh-cut apple continued to decrease except for strawberry treated with L-GRP. GRP coatings could inhibit decreases in its activity from 72 h for strawberry and 12 h for fresh-cut apple (*p* < 0.05). Figures 5G,H show that DPPH• scavenging activities of the two controls remained unchanged at first then decreased (*p* < 0.05) in strawberry but increased (*p* < 0.05) in fresh-cut apple. Nevertheless, both L-GRP and H-GRP coatings finally inhibited their decreases (*p* < 0.05). OH• scavenging activity of the control of strawberry (Figure 5I) kept unchanged first and then decreased (*p* < 0.05), whereas that of the fresh-cut apple (Figure 5J) increased (*p* < 0.05) from 12 h. However, compared to their controls, two GRP coatings onto both the two fruits finally boosted their scavenging rates. The results suggested that GRP coatings can keep fruits' good antioxidant status for a longer time.

#### Effects of GRP coating on polyphenol oxidase activity

As shown in Figure 5K, polyphenol oxidases of both the control and those of GRP-coated fresh-cut apples increased progressively (*p* < 0.05). Nevertheless, both L-GRP and H-GRP were effective in delaying (*p* < 0.05) the increase. Namely, polyphenol oxidases were suppressed (*p* < 0.05) to reduce the browning of the fresh-cut apple. It was worth noting that the polyphenol oxidase activity of strawberry was not detected or was extremely low in this study (data not shown). Polyphenol oxidases are closely related to browning, no obvious browning was observed during the 96h storage of strawberry, which may explain the negative data. This effectiveness was clearly evidenced by appearance images of the strawberry and fresh-cut apple throughout the entire storage duration (Supplementary Figure S2). In addition, several studies on strawberry preservation also did not involve the activity changes of polyphenol oxidases (59, 60).

### Correlation and principal component analyses and possible mechanisms of the preservation effects

#### Correlation analysis of the quality and antioxidant indexes

To evaluate relations among the many fruits' quality and antioxidant parameters measured, a correlation-based approach using the Pearson coefficient was then adopted. As shown in Supplementary Figure S3A, for strawberry, there were strong negative correlations between three such parameters as weight loss, relative electrolyte leakage, and malondialdehyde, and the other parameters as firmness, titratable acid, soluble solids, ascorbic acid, and total phenols as well as DPPH• and OH• scavenging activities that can be associated with taste, flavor, and shelf-life. In this study, GRP coatings could reduce weight loss, relative electrolyte leakage, and malondialdehyde, thus prolonging the shelf-life of strawberry. The correlation of these indexes for the fresh-cut apple (Supplementary Figure S3B) was like that of strawberry (Supplementary Figure S3A); their main difference was reflected by the two indexes of titratable acid and malondialdehyde, which is probably related to the high titratable acid and low malondialdehyde levels in the fresh-cut apple. After all, during our experiment period, strawberry was just harvested, while apple had been stored under normal conditions for at least 4 months.

#### Principal component analyses on quality and antioxidant indexes

**Figure 6:**
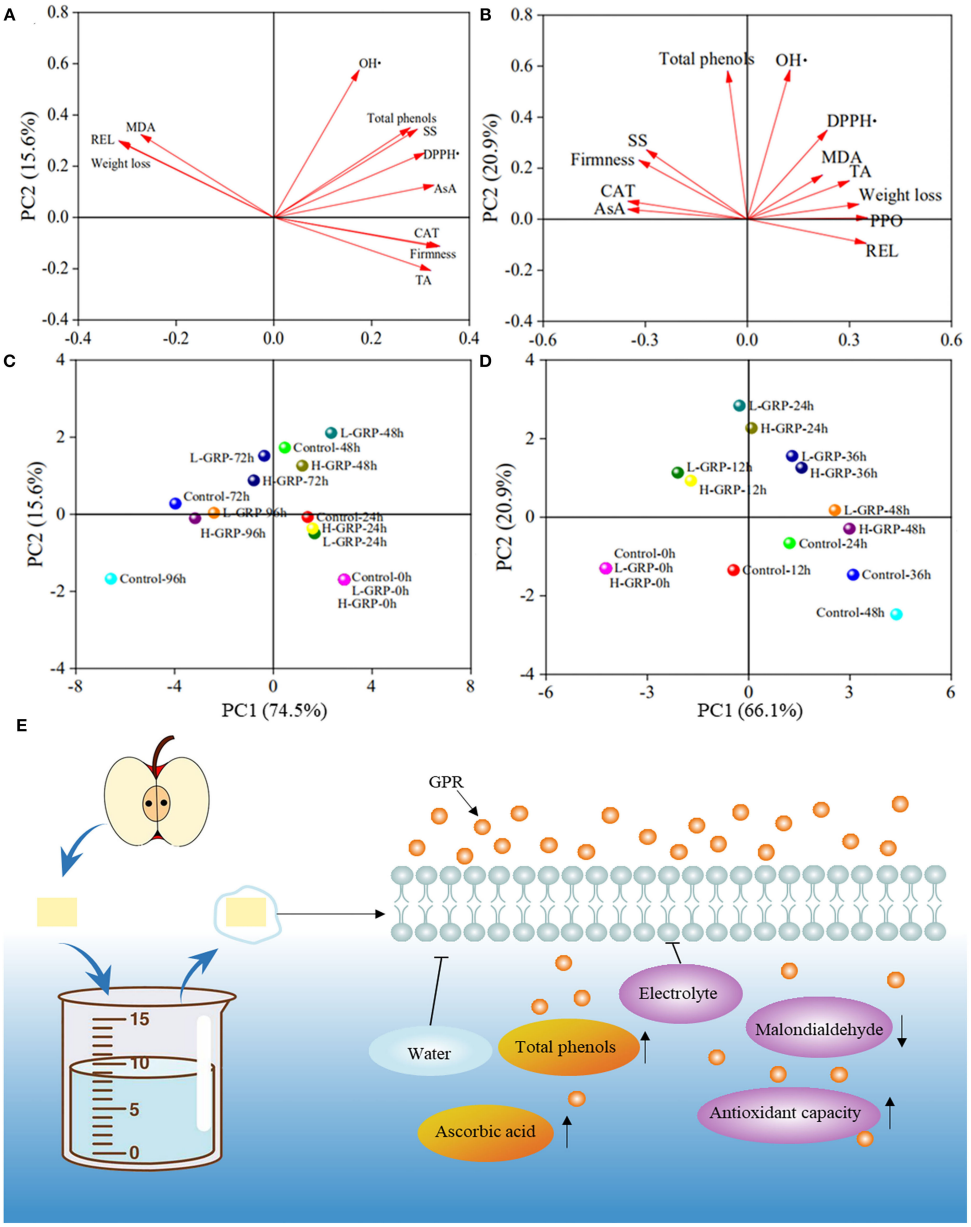
Principal components analyses in loading **(A,B)** and score **(C,D)** plots of different variances in effects of GRP coating on strawberry **(A,C)** and fresh-cut apple **(B,D)** during storage, and taking fresh-cut apple as an example, possible mechanisms **(E)** of GPR coating for fruit preservation. The up and down arrows represent inhibition and boost, respectively. GPR, ginsenosides-extracting residue polysaccharides; AsA, ascorbic acid; CAT, catalase; DPPH•, DPPH radical scavenging activity; MDA, malondialdehyde; OH•, hydroxyl radical scavenging activity; PPO, polyphenol oxidase; REL, relative electrolyte leakage; SS, soluble solids; TA, titratable acidity; Control, fruits treated with deionized water; L-GRP and H-GRP, fruits coated with GRP at 5 and 20 mg/ml in respective. Lengths of arrows **(A,B)** indicate information drawn from a variance; circles of different colors **(C,D)** represent different treatments of strawberry and fresh-cut apple for storage.

Principal component analyses (PCA) were subsequently applied to evaluate the effectiveness of GRP coating at the two different concentrations on both strawberry and fresh-cut apple during their storage. As shown in Figures 6A,B, multivariate treatments of the data obtained for the samples allowed these variables to be reduced to two principal components that explained 90.10 % and 87.00 % of the total variability of strawberry and fresh-cut apple, respectively. A longer arrow of the variance means the PCA can explain more information about that variance (61). In Figure 6A, the length of each arrow was similar, indicating that each index had a similar contribution to PC1 and PC2, whereas in Figure 6B, much information, especially that of malondialdehyde, had been lost to certain extents probably because the PC1 and PC2 only explained 87.00 % of total data variance.

The proximity of different samples on the scoring plots indicated similar behaviors of their effects on the fruit properties (60). The differences between controls and GRP coatings of strawberry (Figure 6C) and fresh-cut apple (Figure 6D) appeared from 48 h and 12 h, respectively, due to the scores between them being different on the score plots. Especially, for strawberry, the control at 72 h and the L-GRP and H-GRP at 96 h were located at the position with similar scores. In addition, for fresh-cut apple, the scores of the control at 36 h were like those of the L-GRP and H-GRP at 48 h. These results confirm that GRP coating showed certain extents of preservation for both strawberry and fresh-cut apple. Interestingly, the scores of L-GRP and H-GRP for strawberry and fresh-cut apple were always similar, indicating that the two GRP coatings exhibited no significant difference in the preservation effect on both the two fruits.

#### Possible mechanisms of GPR coating for fruit preservation

In summary, the above PCA on quality and antioxidant indexes, as well as the correlation analyses within and between the two sets of indexes, can more comprehensively explain the preservation effects of GRP coating on strawberry and fresh-cut apple. The incision to the apple might cause its correlations among the evaluated parameters to be much more complex than those found in the strawberry. Figure 6E, taking fresh-cut apple as an example, outlines a possible mechanism of GRP coating for fruit preservation. A protective coating was formed on the fruit surface when it was soaked in a GRP solution, reducing water evaporation and relative electrolyte leakage, in turn, slowing down the decrease of ascorbic acid and total phenol levels and maintaining the quality of fruit. At the same time, GRP may have permeated into the cell interior through the cell surface and played an antioxidant role, making the formation of malondialdehyde inhibited and the scavenging capacities of DPPH and hydroxyl radicals and the activities of enzymes related to the antioxidant capacity to improve. As a result, GRP-coating extended the shelf-life of the fruit by acting on its surface and interior.

## Conclusions

This study is the first to demonstrate the extraction, purification, and characterization of polysaccharides recovered from ginsenosides-extracting residue and to study their antioxidant activities and preservation performance on strawberry and fresh-cut apple. GRP was extracted and then purified into two fractions, namely GRP-1 and GRP-2. The Mn and Mw of GRP, GRP-1, and GRP-2 were determined. GRP-1 was the main component of GRP, including large starch-like glucans, and a small portion of GRP-2 was composed of starch-like glucans, RG-I pectin, and arabinogalactans. Compared with ginseng root polysaccharides, the contents of RG-I pectin and arabinogalactans of GRP-2 were only slightly decreased. Our results proved that the structure of polysaccharides from ginsenosides-extracting residue was not destroyed during ginsenosides extraction. Nevertheless, the GRP in this study still had a strong antioxidant activity which was probably related to its relatively higher contents of glucuronic and galacturonic acids, since their presence and content are important indexes to reflect the antioxidant activity of polysaccharides. Subsequently, applications of the GRP coating to strawberry and fresh-cut apple for evaluating its effects on the maintenance of fruit quality and nutrition were conducted. The results show that strawberry and fresh-cut apple coated with the GRP delayed the deterioration and maintained nutrients and other quality indicators compared with the control. PCA and correlation analyses on the quality and antioxidant parameters supported the above findings and a possible mechanism for fruit preservation was then proposed.

This study offers a theoretical foundation on the polysaccharides that were recovered from ginsenosides-extraction waste and that has potential and promising antioxidant properties for fruit preservation application. The study will be used as a valuable theoretical reference for future investigation on the development of polysaccharides from plant waste.

## Data availability statement

The original contributions presented in the study are included in the article/Supplementary material, further inquiries can be directed to the corresponding author.

## Author contributions

JS: conceptualization, methodology, investigation, and writing—original draft. XZ: investigation and software. DS and XC: investigation. FY: software. LS: validation. YL: conceptualization, writing—review and editing, and supervision. All authors contributed to the article and approved the submitted version.

## Funding

This research was funded by [Forestry Public Welfare Scientific Research Projects] grant number [201404718], China.

## Conflict of interest

The authors declare that the research was conducted in the absence of any commercial or financial relationships that could be construed as a potential conflict of interest.

## Publisher's note

All claims expressed in this article are solely those of the authors and do not necessarily represent those of their affiliated organizations, or those of the publisher, the editors and the reviewers. Any product that may be evaluated in this article, or claim that may be made by its manufacturer, is not guaranteed or endorsed by the publisher.
