# Supplementary material for: Structural characterization of polysaccharides recovered from extraction residue of ginseng root saponins and its fruit nutrition preservation performance
Source: Front Nutr. 2022 Aug 1;9:934927. doi: 10.3389/fnut.2022.934927 (PMC9376600; doi:10.3389/fnut.2022.934927)
Supplement: Supplementary file 1 [file Image_1.PDF]

Supplementary Material

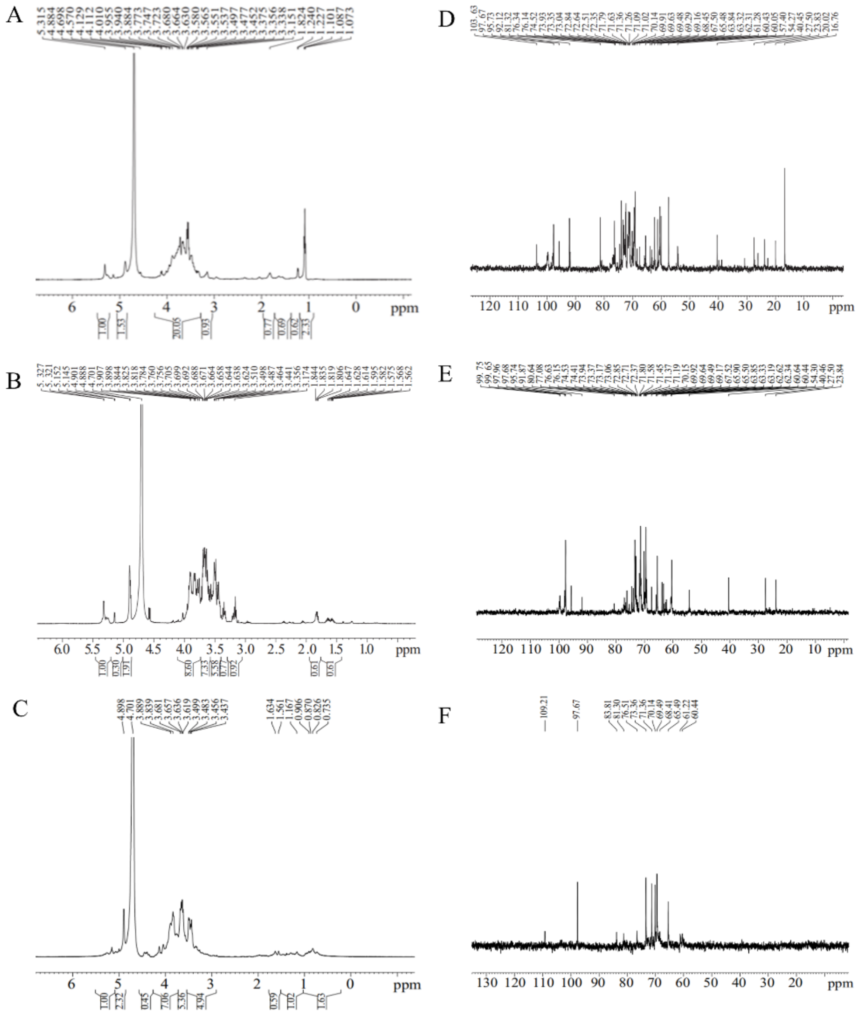

Supplementary Figure 1. Nuclear magnetic resonance spectroscopy (NMR) of GRP, GRP-1 and GRP-2. (**A**), (**B**) and (**C**) were ^{1}H NMR spectra and D, E and F were ^{13}C NMR spectra of GRP, GRP-1 and GRP-2, respectively. GRP, ginsenosides-extracting residue polysaccharides; GRP-1 and GRP-2, fractions separated by 0 M and 0.1 M NaCl from GRP, respectively.

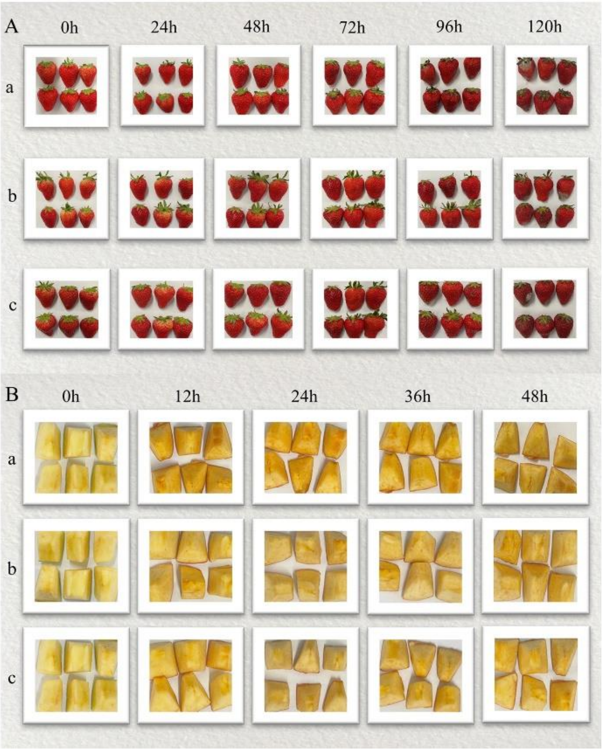

Supplementary Figure 2. Appearance images of strawberry (**A**) and fresh-cut apple (**B**) of the control (**a**) and those treated with L-GRP (**b**) and H-GRP (**c**) at different times. GPR, ginsenosides-extracting residue polysaccharides; Control, fruits treated with deionized water; L-GRP and H-GRP, fruits treated (coated) with GRP at 5 and 20 mg/mL in respective.

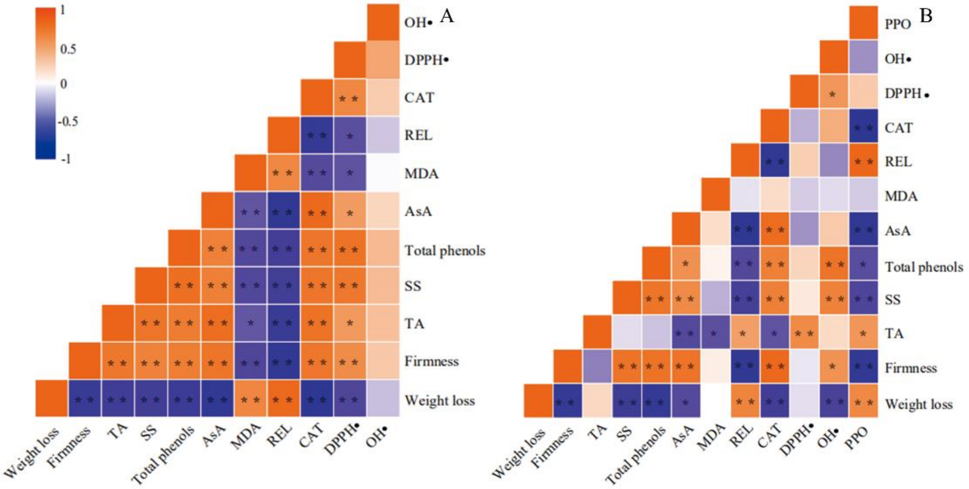

Supplementary Figure 3. Correlation analyses of quality and antioxidant parameters in effects of GRP-coating on strawberry (**A**) and fresh-cut apple (**B**) during storage. Orange and blue colors show the positive and negative correlations, respectively. * and ** indicate significant correlations at $p < 0.05$ and $p < 0.01$, respectively. AsA, ascorbic acid; CAT, catalase; DPPH•, DPPH radical scavenging activity; MDA, malondialdehyde; OH•, hydroxyl radical scavenging activity; PPO, polyphenol oxidase; REL, relative electrolyte leakage; SS, soluble solids; TA, titratable acidity.
